# List of Hospitals and Medical Schools

**Published:** 1892-09-24

**Authors:** 


### List of Hospitals and Medical Schools.

I.—Universities and Corporations which both Educate Students and Confer Degrees.

The University of Oxford, M.B., M.D., and B.S.
The University of Cambridge, M.B., M.D., and B.S.
The University of Durham, M.B., M.D., and C.M.
Victoria University, Manchester, M.B., M.D., and C.M.
The University of Edinburgh, M.B., M.D., and C.M.
The University of Glasgow, M.B., M.D., and C.M.
The University of Aberdeen, M.B., M.D., and C.M.
The University of St. Andrew's, M.B., M.D., and C.M.
Trinity College, Dublin, M.B., M.D., and C.M.

II.—University and Corporations which Grant Degrees, but have no Medical School Attached.

The Royal University of Ireland, M.B. and M.D.
University of London, M.B. and M.D.
The Royal College of Physicians, London, L.R.C.P., M.R.C.P.
The Royal College of Surgeons, London, M.R.C.S.
The Apothecaries Society, London, L.S.A.
The Royal Colleges of Physicians and Surgeons, Edinburgh, L.R.C.P. and L.R.C.S.
The Faculty of Physicians and Surgeons, Glasgow, L.F.P. and S.G.
The King's and Queen's Colleges of Physicians, Ireland. L.K.Q.C.P.I.
Royal College of Surgeons, Ireland, L.R.C.S.I.

III.—Hospitals to which Medical Schools are Attached but which do not Confer Practising Qualifications.

1.—*London Hospitals.*

St. Bartholomew's Hospital.
Charing Cross Hospital.
Guy's Hospital.
King's College Hospital.
The London Hospital.
Middlesex Hospital.
St. George's Hospital.
St. Mary's Hospital.
St. Thomas's Hospital.
University College Hospital.
Westminster Hospital.
London School of Medicine for Women.
The College of State Medicine.

2.—*Provincial Hospitals.*

Bristol General Hospital.
Birmingham General Hospital.
Leeds General Hospital.
Sheffield General Hospital.
Manchester General Hospital.
Newcastle-on-Tyne (University of Durham) General Hospital.

3.—*Scottish Hospitals.*

Edinburgh General Hospital.
Glasgow Royal Infirmary.
Glasgow Western Infirmary.
Aberdeen General Hospital.
Dundee Royal Infirmary (St. Andrews University).

4.—*Irish Hospitals.*

The Rotunda (Midwifery).
Adelaide Hospital.
Catholic University Hospital.
City of Dublin Hospital.
Coombe Lying-in Hospital.
Mater Misericordiae Hospital.
Meath Hospital.
Queen's College Hospital, Belfast.
Queen's College Hospital, Cork.
Queen's College Hospital, Galway.

For information required about any of these universities, hospitals, and their medical schools, it is sufficient to write to the Dean of the school or the Secretary at the hospital.

